# Supplementary material for: Complex‐centric proteome profiling by SEC‐SWATH‐MS
Source: Mol Syst Biol. 2019 Jan 14;15(1):e8438. doi: 10.15252/msb.20188438 (PMC6346213; doi:10.15252/msb.20188438)
Supplement: Supplementary file 7 — Dataset EV6 [file MSB-15-e8438-s007.zip › feature_plots_bioplex/P35998.pdf]

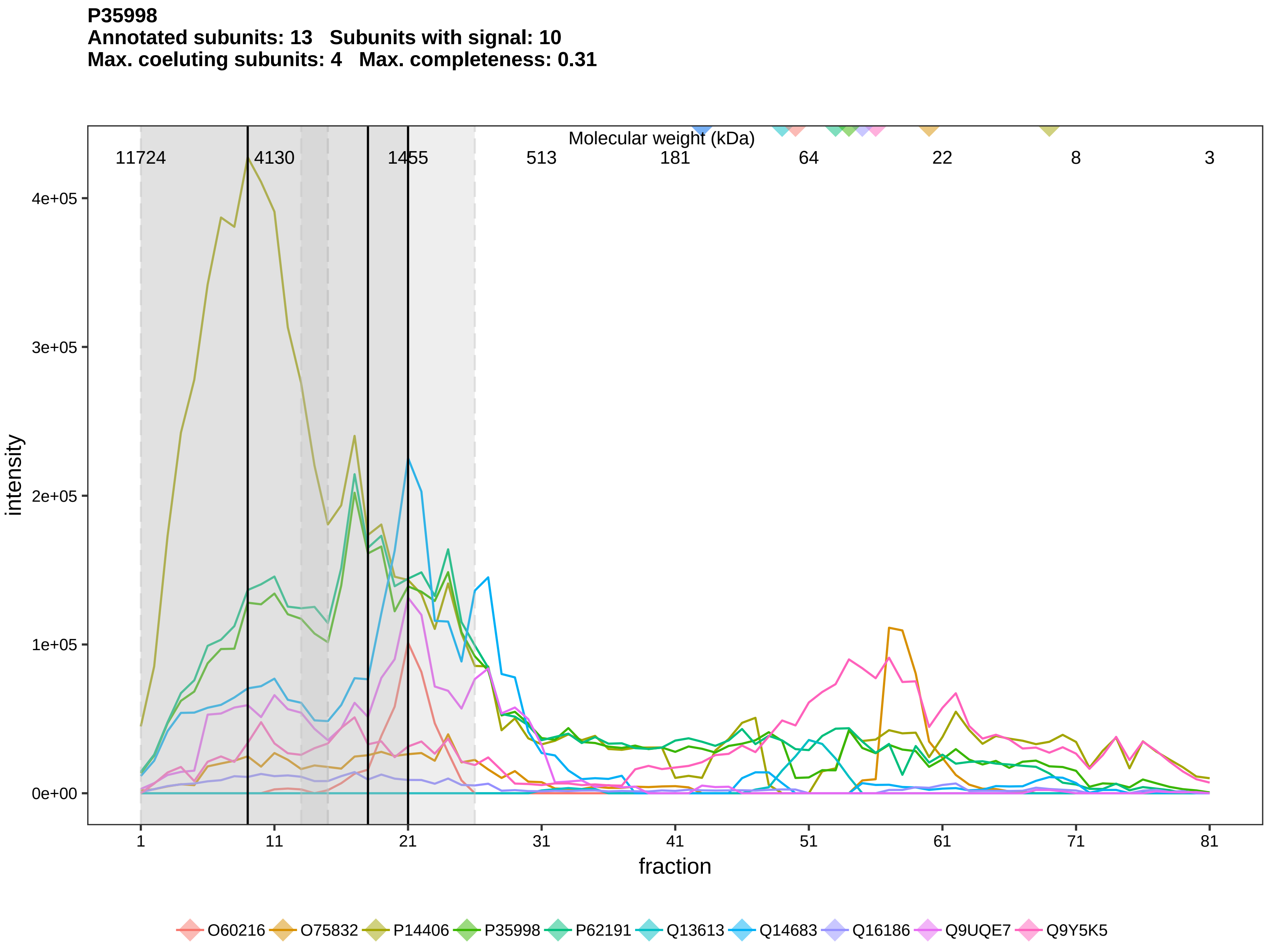
P35998
Annotated subunits: 13 Subunits with signal: 10
Max. coeluting subunits: 4 Max. completeness: 0.31
Molecular weight (kDa)
11724
4130
1455
513
181
64
22
8
3
4e+05
3e+05
2e+05
1e+05
0e+00
intensity
1
11
21
31
41
51
61
71
81
fraction
O60216
O75832
P14406
P35998
P62191
Q13613
Q14683
Q16186
Q9UQE7
Q9Y5K5
